# Causes of Low Milk Supply: The Roles of Estrogens, Progesterone, and Related External Factors

**DOI:** 10.1016/j.advnut.2023.10.002

**Published:** 2023-10-11

**Authors:** Xuehua Jin, Sharon L. Perrella, Ching Tat Lai, Nicolas L. Taylor, Donna T. Geddes

**Affiliations:** 1 School of Molecular Sciences, The University of Western Australia, Crawley, Western Australia, Australia; 2 ARC Training Centre in Biomedical Analysis, The University of Western Australia, Crawley, Western Australia, Australia

**Keywords:** breastfeeding, lactation, low milk supply, obesity, estrogen, progesterone, phytoestrogen, mycoestrogen, endocrine-disrupting chemicals, contraceptive

## Abstract

Low milk supply (LMS) poses a significant challenge to exclusive and continued breastfeeding, affecting ∼10% to 15% of mothers. Milk production is intricately regulated by both endocrine and autocrine control mechanisms, with estrogens and progesterone playing pivotal roles in this process. In addition to endogenously produced hormones, external substances capable of interfering with normal hormonal actions, including phytoestrogens, mycoestrogens, synthetic estrogens, and hormonal contraceptives, can influence milk production. The effects of these extrinsic hormones on milk production may vary based on maternal body mass index. This comprehensive review examines the multifaceted causes of LMS, focusing on the involvement of estrogens, progesterone, and related external factors in milk production. Furthermore, it investigates the interplay between hormonal factors and obesity, aiming to elucidate the endocrine mechanisms underlying obesity-associated LMS. Insights from this review provide valuable perspectives for developing interventions to improve milk production and address the challenges associated with LMS.

**Statement of Significance:**

This review constitutes a pioneering effort to integrate endogenous and exogenous hormonal factors, providing valuable insights into the complex endocrine mechanisms associated with low milk supply in the context of obesity. Particular emphasis is placed on the significance of hormone production and accumulation within adipose tissue.

## Introduction

Human milk is the optimal food for infants, providing nutritional, immunologic, and developmental advantages that extend into childhood and adulthood [1,2]. WHO and UNICEF recommend that mothers initiate breastfeeding within 1 h of giving birth, exclusively breastfeed for the first 6 mo, and continue breastfeeding ≤2 y and beyond [3]. However, as reported by UNICEF global data for 2015–2021, only 47% of infants were breastfed within an hour of birth, while 48% were exclusively breastfed ≤5 mo. Additionally, only 65% of infants, irrespective of their exclusive breastfeeding status, continued to be breastfed at 12–23 mo [4]. This illustrates that there are still persistent and significant barriers to successful breastfeeding. A variety of reasons for terminating breastfeeding are reported by mothers, among which perceived low milk supply (LMS) is one of the most common [[5], [6], [7]]. Actual LMS is believed to occur in ∼10% to 15% of mothers, and due to the increasing prevalence of obesity and diabetes, which is associated with LMS, this rate is likely to be higher [8,9]. Hence, identifying and addressing the underlying biochemical factors that contribute to LMS is crucial to increasing breastfeeding rates.

Milk production is controlled at 2 levels. At the systemic level, signals are transmitted to the mammary gland via the endocrine system, while local control or autocrine regulation occurs through a feedback control system in response to components in the milk [10]. The complex interplay of these regulatory mechanisms involves multiple hormones, including estrogens and progesterone, 2 key reproductive hormones that play a critical role in mammary gland development [11,12]. Aside from the naturally occurring estrogens and progesterone, external chemicals that interfere with normal hormone actions can also affect milk production [13,14]. Mothers are exposed to these external factors consciously or unconsciously through their daily diet, personal care products, plastics, packaging materials, pesticides, and pharmaceuticals, such as ethinylestradiol, cimetidine, and fenofibrate [15,16]. Although most external chemicals are not as potent as endogenous hormones [17], due to the continuous exposure and persistent accumulation of external chemicals [18], their roles in milk production are worthy of investigation.

In this review, we will provide an overview of the causes of LMS and examine the roles of estrogens, progesterone, and related external factors in milk production. This review aims to explore potential endocrine explanations for LMS and provide insights into developing effective interventions for improving milk production and supporting breastfeeding.

## Causes of LMS

### Extrinsic causes

Extrinsic causes of LMS include those that limit the removal of milk from the breast. Frequent and effective milk removal plays a vital role in the development of robust milk production, with early initiation of infant suckling recognized as one determinant of successful breastfeeding [19,20]. Therefore, some infant conditions and behaviors that affect suckling ability are known to have a major impact on milk removal. Preterm infants with immature suckling skills and infants who have undergone prolonged labor may be too weak to latch to the breast and suckle effectively [21]. Anatomic abnormalities that impede suckling, such as cleft palate and ankyloglossia, as well as congenital disorders affecting infant behaviors, such as hypothyroidism, Down syndrome, and neurologic disturbances, can also impact milk removal and stimulation of the breast causing a delay in the onset of lactation [2]. Further, feeding practices such as time-limited feeds and feeding schedules that reduce breastfeeding frequency can result in inadequate milk removal with subsequent downregulation of milk production [22].

Importantly, sociocultural factors must be considered as some cultural beliefs and lack of breastfeeding knowledge, prelacteal feeding, or feeding of substances other than breast milk in the days after birth is widespread across the world, especially in low- and middle-income countries [23]. Prelacteal feeding is associated with delayed secretory activation and shorter breastfeeding duration [24]. Furthermore, advertising of commercial milk formula products has increased their popularity and use, leading some mothers to introduce formula supplementation as they believe it to be equal to or better than breast milk [25,26]. With both prelacteal and formula feeding, milk removal from the breast through breastfeeding is reduced, thereby reducing maternal milk production via autocrine control.

LMS can also be attributed to altered breast anatomy resulting from external factors, including previous breast surgery and nipple piercing, as these procedures disrupt the milk ducts and consequently hinder milk removal [[27], [28], [29]]. Moreover, shorter lactation duration and LMS have been observed in smoking mothers [[30], [31], [32]]. Although there is a debate as to whether this is a result of lower breastfeeding motivation or a physiologic effect [33], it has been reported that nicotine from cigarettes can reduce maternal prolactin concentrations and affect the normal suckling activities of infants [34,35]. Further, recent research indicates that maternal exposure to endocrine-disrupting chemicals (EDCs) is associated with lower rates of breastfeeding initiation and shorter breastfeeding duration [13,36]. Numerous EDCs have been found to impact the development or functions of key organs involved in lactation, including the pituitary gland, mammary gland, and placenta [11].

### Intrinsic causes

Intrinsic causes of low milk production include developmental and endocrine conditions that alter glandular tissue development and function. Breast hypoplasia is a primary reason for an inherent inability to produce sufficient milk [37]. Features of hypoplasia include a wide intermammary space, breast asymmetry, and a tubular shape of the breasts [38]. Women with hypoplasia may not experience breast development during pregnancy and lack sufficient glandular tissue to produce enough milk to meet their newborn infant’s needs [37]. At the genetic level, zinc transporter mutations are linked to altered mammary gland development and function during lactation [39,40]. For example, Thr288Ser mutation in ZnT2 (SLC30A2) has been identified in a sample of women with LMS. This mutation alters lysosome function and cell energetics, thus impairing critical functions of the mammary epithelial cells (MECs) [41].

Increasing evidence also suggests that maternal metabolic disorders are associated with LMS. Gestational diabetes mellitus (GDM) is the most common pregnancy complication and is increasing in prevalence globally [42]. A few studies have found that women with GDM are at greater risk of delayed secretory activation and LMS [[43], [44], [45]]. Insulin regulates the expression of genes involved in milk protein synthesis, including signal transducer and activator of transcription 5 (STAT5a) and E74-like factor 5, which are key components of prolactin signaling [46,47]. Therefore, insulin resistance might be a potential mechanism underpinning the link between GDM and LMS that has been shown in a recent case series [43]. There is no doubt that pre-existing type 1 and type 2 diabetes mellitus (T1DM and T2DM) are also associated with poor breastfeeding outcomes [48]. As compared with women without diabetes, women with T1DM had lower breastfeeding rates and shorter breastfeeding duration [49]. Moreover, delayed secretory activation was observed in women with T2DM as well as lower rates of exclusive breastfeeding at 4 mo postpartum [50]. In addition to diabetes, women with polycystic ovary syndrome (PCOS) may have lower rates of breastfeeding exclusivity and duration [51,52]. PCOS affects 9%–21% women of reproductive age, and key features of insulin resistance and androgen excess might explain the hormonal mechanisms by which PCOS may impair lactation [53,54]. However, one study indicated that the suboptimal breastfeeding outcomes associated with PCOS are related to maternal obesity rather than PCOS status per se [55], with obesity identified in ≤88% of women with PCOS [56].

Obesity is widely recognized as a risk factor for a range of metabolic disorders, including GDM, T2DM, and PCOS [[56], [57], [58]]. Additionally, obesity itself can affect breastfeeding and be a contributing factor for LMS. Compared to women who have a normal BMI, women who are overweight or obese are less likely to initiate, exclusively breastfeed, or continue breastfeeding [59,60]. Women with obesity may have reduced ductal branching and impaired alveolar development as a negative consequence of increased fat deposition, similar to that observed in rodent studies [61]. Adipose tissue is a significant peripheral source of aromatase activity in women, and steroid hormones are produced and stored in adipose tissue [62,63]. Coupled with the fact that local estrogen production is correlated with obesity [64, 65], Knight [66] hypothesized that local estrogen production by aromatization might account for the shorter breastfeeding duration observed in women with obesity. It is also postulated that these women may have a delayed decline in blood progesterone concentration after birth as a result of the progesterone produced or stored in excess adipose tissue [[66], [67], [68]]. Another potential reason for the shorter breastfeeding duration associated with obesity may be pervasive exposure to EDCs [15,36,69]. These EDCs are known to alter endocrine regulation by acting directly through cellular steroid receptors within the mammary gland or by influencing the synthesis of estrogens [11]. Darbre [70] has suggested a potential vicious cycle between obesity and EDCs, whereby EDCs act as obesogens and increase the amount of body fat, which in turn results in greater retention of lipophilic EDCs. The greater amount of adipose tissue in large breasts may produce or store higher levels of endogenous steroid hormones and environmental lipophilic chemicals, ultimately exerting an adverse impact on milk production. Moreover, health care providers have reported that when compared to women with obesity and women with large breasts, women with both obesity and large breasts face greater challenges in initiating breastfeeding [71]. Some studies have shown that women with large breasts find it more difficult to position their infants for breastfeeding [72,73]. Although the suboptimal breastfeeding outcomes associated with obesity are likely multifactorial, evidence of endocrine disruption deserves further investigation.

**Figure 1:**
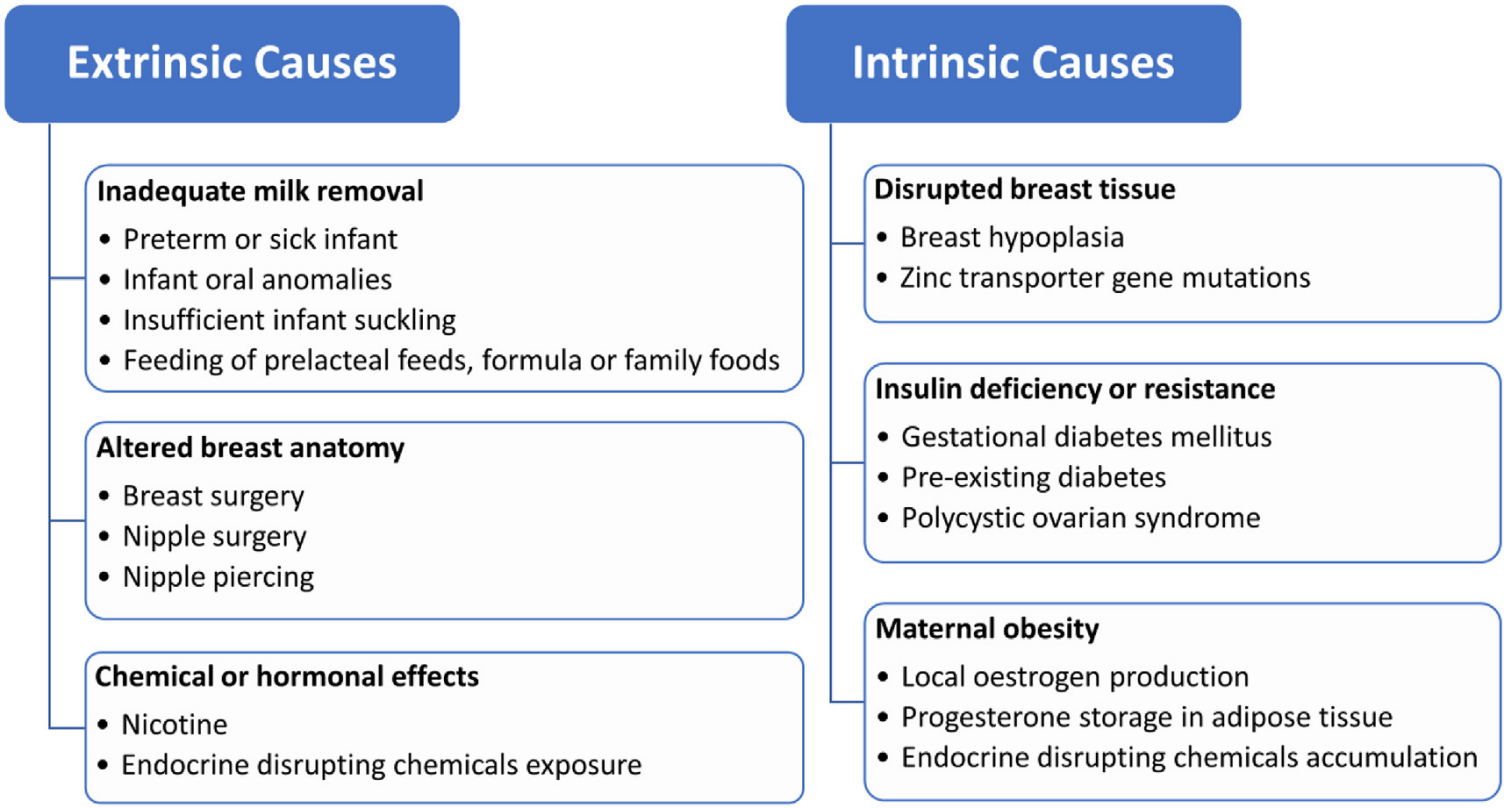
Potential causes of low milk supply.

Figure 1 integrates the causes of LMS. The remainder of the review will discuss the roles of estrogens, progesterone, and related external factors affecting milk production, thereby supporting the endocrine explanations for obesity-associated LMS.

## The roles of estrogens and progesterone in milk production

### Estrogens

Estrogens are a type of 18-carbon steroid that derive from cholesterol and are predominantly produced by the ovaries and placenta [74]. The 3 most common endogenous estrogens are estrone (E1), 17β-estradiol (E2), and estriol (E3), with E2 being the most potent and extensively studied form [75]. Although E1 and E3 can interact with estrogen receptors, their binding affinity is typically lower [76], resulting in limited research attention concerning their implications for lactation. As summarized in Table 1, E2 is involved in the development of the breast during pregnancy by stimulating prolactin secretion from the anterior pituitary and increasing prolactin receptor expression in the mammary epithelium [[77], [78], [79]]. It has been shown that E2 promotes lipid formation in mammary epithelium cells via the regulation of lipid synthesis enzymes [80,81]. Nevertheless, during the subsequent lactation period, estrogens exert a suppressive effect on milk production. According to a prospective observational study of 91 women, there was a negative association between plasma E2 concentration and milk output at 4 wk postpartum [82]. It was also demonstrated in bovine studies that E2 injection decreased milk production and accelerated involution during the drying off period [83,84]. If a high concentration of E2 persists for long periods of time, the mammary tight junctions (TJs) can be disrupted and result in the transfer of lactose from milk to plasma or urine [84]. Additionally, E2 enhances apoptotic processes in bovine MECs, and this may contribute to the negative effects on milk production [85]. After weaning, E2 promotes mammary gland involution by increasing inflammation, cell death, and adipocyte repopulation, which was described in a mice model [86].

**TABLE 1:** The roles of estradiol and progesterone in lactation

Hormone	Stage	Study model	Effects on lactation Negative (−) or Positive (+) or No effects (×)		Refs.
Estradiol	Pregnancy	Mouse	• Stimulates MECs to promote alveologenesis	+	[78]
		*In vitro*	• Mammary epithelium proliferation	+	[79]
		*In vitro*	• Promotes lipid formation in MECs	+	[80,81]
		Human	• Ensures adequate prolactin secretion and permits lactogenesis in response to prolactin	+	[77]
	Established lactation	Cow	• Decreases milk production	−	[83]
		Cow	• Decreases milk production	−	[84]
			• Increases lactose in plasma and urine		
			• Loss of mammary TJ integrity		
		*In vitro*	• Enhances apoptotic processes in MECs	−	[85]
	Postweaning	Cow	• Accelerates mammary gland involution	−	[83]
		Mouse	• Increases inflammation, cell death, and adipocyte repopulation	−	[86]
Progesterone	Pregnancy	Mouse	• Mammary epithelium proliferation	+	[91]
		Mouse	• Ductal morphogenesis	+	[92]
		*In vitro*	• Promotes lipid formation in MECs	+	[80, 81]
		*In vitro*	• Blocks glucocorticoid receptors in mammary tissue	−	[93]
		Rabbit	• Blocks the ability of prolactin to increase the number of prolactin receptors in the mammary gland	−	[94]
		Rabbit	• Prevents casein mRNA translation	−	[95]
		*In vitro*	• Regulates lactose synthesis and acts to repress the formation of α-lactalbumin throughout pregnancy	−	[96]
	1–4 d postpartum		Progesterone withdrawal triggers:		
		Human	• secretory activation	+	[97,98]
		Mouse	• mammary TJ closure	+	[99]
		Human	• prolactin receptor signaling via STAT5	+	[100]
	Established lactation	Rat	• Inhibits apoptosis in the presence of the normal suckling stimulus	+	[101]
		Rat	• No effects on milk production	×	[102]

Abbreviations: MEC, mammary epithelial cell; STAT, signal transducer and activator of transcription; TJ, tight junction.

Estrogens are also produced in extragonadal sites and act locally as paracrine or even intracrine factors [87]. Studies have shown that adipose tissue contains high levels of the estrogen-metabolizing cytochrome P450 enzymes, which allow the conversion of androgens to estrogens [88]. Hence, local estrogen production within mammary adipose tissue could potentially explain LMS in women with obesity or other obesity-related metabolic disorders [66]. In fact, one study found that women with prepregnancy overweight/obesity had a delayed decline in serum E2 concentration at 48 h postpartum, which was correlated with delayed onset of secretory activation [89].

### Progesterone

Progesterone is a 21-carbon steroid hormone made from cholesterol. During the menstrual cycle the majority is produced by the corpus luteum in the ovary, whereas during pregnancy the placenta is the main source of progesterone [90]. Antenatal serum progesterone levels have a positive association with postnatal milk production [82], because they promote mammary epithelium proliferation and ductal morphogenesis during pregnancy [91,92]. At the same time, however, progesterone blocks lactogenesis in the antenatal period, due to its suppression of prolactin and glucocorticoid receptors in mammary tissue [93,94]. Moreover, it was also reported that progesterone inhibits the synthesis of α-lactalbumin, casein, and lactose, thereby inhibiting the onset of lactogenesis [95,96]. It is not until after the placenta is delivered following birth that progesterone concentration declines rapidly, which triggers the onset of secretory activation [97,98]. The immediate withdrawal of progesterone also triggers the closure of mammary TJs and prolactin receptor signaling via STAT5, which are required for copious milk production [99,100]. Additionally, progesterone appeared to inhibit apoptosis in the lactating rat mammary gland in the presence of normal suckling stimulus [101]. Despite this, progesterone is shown to have little effect on milk production. This is evident from studies conducted on rats, where the administration of 2 mg of progesterone injections during the first or second week after parturition did not markedly affect lactation [102].

Fehér et al. [103] reported that the human adipose tissue: serum concentration ratio of progesterone is 6.3. This ratio is even higher in individuals with obesity, reflecting the significantly higher concentration stored in adipose tissue. As a bovine study has shown substantial progesterone to be sequestrated in adipose tissue that may not be released at parturition [104], it has been speculated that progesterone sequestration in adipose tissue might impair the onset of secretory activation in women with obesity [66]. However, studies comparing serum progesterone concentrations between prepregnant obese and healthy BMI groups found no significant differences in the rate of decline of serum progesterone concentrations from 37 wk of gestation to 48 h postpartum and from 48 h to 7 d postpartum [89,105]. Nonetheless, this hypothesis merits further investigation with larger sample sizes and sequential sampling across the early postpartum period. Another aspect to consider is that serum is not the sole pathway for progesterone excretion. During lactation, progesterone stored in adipose tissue can also be released through breast milk, given its high affinity for milk fat [106]. To date, no studies have reported a comparison of human milk progesterone concentrations between groups of women with normal, overweight, and obese BMI classifications.

## External factors affecting milk production

### Phytoestrogens

Phytoestrogens, also known as “dietary estrogens,” are estrogenic compounds found in various plant-based sources, including legumes, flaxseed, fruits, vegetables, and cereals [107]. They are classified into 3 main classes: isoflavones, coumestans, and lignans [108]. Due to their structural similarity to E2, these polyphenolic compounds can interact with estrogen receptors, exerting estrogenic or antiestrogenic activities in the body [109]. Investigations into the impact of phytoestrogens on milk production have primarily relied on animal models and *in vitro* data. Most attention has been devoted to isoflavones, including biochanin A, formononetin and their metabolites genistein, daidzein, p-ethylphenol, and equol. Tsugami et al. [[110], [111], [112], [113]] and Kumai et al. [114] have demonstrated using mouse and cow models that isoflavones have different effects on mammary gland development, TJ formation, and expression of milk components, thereby affecting milk production (Table 2). Specifically, biochanin A and its metabolite genistein have been shown to inhibit mRNA expression and secretion of both β-casein and lactoferrin [110,112]. Biochanin A also reduced activated STAT5 and increased activated STAT3, which are the negative indicators for milk component production and TJ barriers [110]. Genistein can induce apoptosis in MECs and alter the expression of TJ proteins, leading to weakened barrier function [113]. Furthermore, formononetin had an adverse effect on milk production by decreasing intracellular and secreted β-casein and increasing activated STAT3 [110,112]. In contrast, the effects of daidzein remain uncertain. Most studies suggested that daidzein had no inhibitory effects on milk production [[110], [111], [112],^114^], whereas one study indicated its potential to weaken TJ barriers [113]. Regarding secondary metabolites, both p-ethylphenol and equol have been associated with positive effects on milk production [[110], [111], [112]]. It appears that inhibitory actions of the upstream isoflavones may be rendered ineffective through metabolic conversion by intestinal bacteria [112]. Apart from isoflavones, coumestrol has also been the focus of some research with in vitro studies using mouse MECs showing that coumestrol has an inhibitory effect on milk production, similar to the effects observed with biochanin A and genistein [[112], [113], [114]].

**TABLE 2:** The roles of exogenous hormones in lactation

Exogenous hormones		Study model	Effects on lactation Negative (−) or Positive (+) or No effects (×)		Refs.
Phytoestrogens	Biochanin A	*In vitro*	• Decreases intracellular and secreted β-casein	−	[110,112]
			• Downregulates mRNA expression of whey acidic protein, lactoferrin and α-lactalbumin		
			• Decreases activated STAT5 and increased activated STAT3		
	Genistein	*In vitro*	• Inhibits ductal branching and alveolar formation	−	[[111], [112], [113], [114]]
			• Induces apoptosis in MECs		
			• Decreases intracellular and secreted β-casein		
			• Downregulates mRNA expression of whey acidic protein, lactoferrin, and α-lactalbumin		
			• Downregulates expression of prolactin receptor and STAT5, accompanied by a decrease in STAT5 phosphorylation		
			• Changes expression of TJ proteins, weakens barrier function		
	p-ethylphenol	*In vitro*	• No effects on bovine MECs	×	[110]
			• Upregulates β-casein and whey acidic protein	+	[112]
	Formononetin	*In vitro*	• Increases activated STAT3	−	[110,112]
			• Decreases intracellular and secreted β-casein		
	Daidzein	*In vitro*	• Increases claudin-3 [the main component for less-permeable TJs in lactation]	+	[110,112]
			• Increases activated STAT5		
			• Upregulates β-casein, whey acidic protein, and α-lactalbumin		
			• Does not inhibit milk production	×	[111,114]
			• Weakens TJ barrier function	−	[113]
	Equol	*In vitro*	• Increases claudin-3	+	[[110], [111], [112]]
			• Increases activated STAT5		
			• Decreases activated STAT3		
			• Upregulates β-casein, whey acidic protein, and α-lactalbumin		
	Coumestrol	*In vitro*	• Inhibits ductal branching and alveolar formation	−	[[112], [113], [114]]
			• Decreases intracellular and secreted β-casein		
			• Downregulates mRNA expression of whey acidic protein, lactoferrin, and α-lactalbumin		
			• Downregulates expression of prolactin receptor and STAT5 accompanied by a decrease in STAT5 phosphorylation		
			• Changes expression of TJ proteins and weakens TJ barrier function		
			• Induces apoptosis in MECs		
Mycoestrogens	ZEN	Human	• Delays onset of secretory activation	−	[124]
		Rat	• Enhances damage and toxicity caused by AFB1	−	[125]
			• Coexposure with AFB1 reduces lactation capacity		
Nonpersistent synthetic estrogens	BPA	Rat	• Delays mammary gland alveolar maturation during secretory activation	−	[134]
			• Modifies synthesis and secretion of milk fat, altered milk lipid content, and fatty acid composition		
		Rat	• Delays mammary gland differentiation	−	[135]
			• Lower milk production		
			• Modifies milk protein synthesis and secretion		
		Human	• Increases risk of early breastfeeding termination	−	[132,133]
	BPS	Mouse	• Alters mammary gland histoarchitecture	−	[136]
			• Alters prolactin signaling		
			• Alters expression of estrogen receptors		
			• Causes difficulties in initiating breastfeeding		
	Phthalates	Rat	• Impaired mammary gland development	−	[137]
		Human	• No significant association with breastfeeding exclusivity or duration	×	[138]

Abbreviations: AFB1, aflatoxin B1; BPA, bisphenol A; BPS, bisphenol S; MEC, mammary epithelial cell; STAT, signal transducer and activator of transcription; TJ, tight junction; ZEN, zearalenone.

Interestingly, phytoestrogens seem to have a beneficial impact on obesity, with reduced weight and adipose tissue observed in mice exposed to phytoestrogens [115]. However, results of clinical studies on the influence of phytoestrogens on human body composition and the prevalence of obesity are inconsistent [116]. It is suggested that the effects of phytoestrogens on adipogenesis are dose-dependent, with inhibition of adipogenesis at low doses and stimulation of adipogenesis at high doses [117]. Moreover, the presence of specific intestinal bacteria capable of producing bioactive metabolites is another important factor [118,119]. For instance, in individuals who do not produce equol, overweight and obesity are more common, and supplementation with isoflavones is less effective in improving serum glucose and low-density lipoprotein cholesterol concentrations [120]. Overall, considering that obesity is one of the risk factors for LMS, harnessing the beneficial properties of phytoestrogens to alleviate obesity may have the potential to positively influence milk production.

### Mycoestrogens

Mycoestrogens are secondary fungal metabolites that can mimic natural estrogens by acting as ligands for estrogen receptors [121]. One of the most prevalent mycoestrogens is zearalenone (ZEN), primarily produced by the Fusarium species. ZEN exhibits a strong binding affinity to estrogen receptors due to its structural similarity to E2 [122]. Despite the prevalence of ZEN exposure among reproductive-age women [123], there are limited reports on its effects on milk production. Memiş et al. [124] has reported that mothers with delayed onset of secretory activation were likely to have higher ZEN levels in their breast milk, suggesting ZEN exposure may contribute to lactation initiation difficulties. Another study using a rat model demonstrated that coexposure to ZEN and aflatoxin B1 (AFB1) could reduce lactation capacity, with ZEN enhancing the damage and toxicity caused by AFB1 [125]. This might be attributed to the ability of ZEN to impact various sex hormone concentrations by altering the function of reproductive organs, such as the ovary, uterus, and placenta [126]. However, it has also been shown that low-dose ZEN partially alleviated the damage caused by AFB1 [125], possibly due to its promotion of cell proliferation and metabolism to repair damaged cells [127].

From the limited studies available, it appears that obesity can influence the toxicity of ZEN. González-Alvarez et al. [128] found that obesity acted additively with ZEN-induced toxicity in ovaries. Another study reported an increase in the concentration of serum-free ZEN with an increase in BMI [123]. This occurred despite a decline in total ZEN and a trend to decreased circulation of ZEN conjugates. Therefore, mothers with obesity are more likely to convert ZEN into free forms, which are considered more biologically active and potent than conjugated forms [129].

### Synthetic estrogens

The vast majority of what are commonly referred to as EDCs are synthetic estrogens, which are widely used in everyday consumer products and industrial manufacturing processes [130]. They can be categorized as persistent and nonpersistent chemicals. Persistent organic pollutants (POPs), listed in the Stockholm Convention, are known for their significant threats to human health and so have been subjected to elimination or restriction in production [131]. These POPs have been shown to have adverse effects on mammary gland development and lactation. Criswell et al. [36] summarized that per- and polyfluoroalkyl substances can reduce breastfeeding duration by impairing lactogenesis and suppressing endocrine signaling, while the associations of halogenated aromatic hydrocarbons and organochlorine pesticides with breastfeeding duration have been modest or equivocal in epidemiologic studies. Besides POPs, emerging evidence suggests that nonpersistent chemicals may also influence milk production (Table 2). Recent observational studies have found a link between high exposure to bisphenol A (BPA) during pregnancy and shorter breastfeeding duration [132,133]. Rodent studies have revealed that offspring of BPA-exposed rats experienced delayed mammary gland differentiation and alveolar maturation as well as alterations in milk protein and fat compositions [134,135]. Similarly, pups treated with bisphenol S were less likely to initiate lactation [136]. Further, toxicologic research has indicated that phthalates may impair mammary gland development through estrogenic mechanisms [137]. However, one study involving 725 women found no association between 9 maternal pregnancy urinary phthalate metabolites and the duration of exclusive or any breastfeeding [138].

As previously mentioned, the obesogenic effects of synthetic estrogens contribute to the accumulation of adipose tissue, which in turn can act as a reservoir for enhanced retention of lipophilic chemicals [15,70]. Considering the established associations between obesity and metabolic disorders, it is plausible that synthetic estrogens may also be implicated in maternal metabolic disorders [139]. This is supported by epidemiologic evidence linking high dioxin levels to an increased risk of diabetes and altered glucose metabolism [140]. Collectively, the impact of synthetic estrogens on milk production can occur through direct impairment of mammary gland development or indirect effects on maternal metabolic systems. Further research is warranted to investigate the influence of various synthetic estrogen exposures across different BMI groups and their implications for lactation outcomes.

### Hormonal contraceptives

In addition to dietary or environmental intake of exogenous hormones, breastfeeding mothers may also use hormonal forms of contraception that contain estrogens and/or progesterone. Hormonal contraceptives are generally classified as progestin-only contraceptives (POCs) administered orally, by injection, implant, or intrauterine device, and combined hormonal contraceptives (CHCs) administered orally. Their effects on milk production have been systematically summarized in recent studies [[141], [142], [143], [144]]. Although the findings are not entirely consistent, the overall weight of evidence suggests that POCs have no detrimental impact on breastfeeding outcomes [142,144]. It is recommended to initiate POCs after the onset of secretory activation considering potential adverse effects on lactation as well as the risk of intrauterine device expulsion and prolonged vaginal bleeding [142,145]. In the case of CHCs, some studies have reported decreased breastfeeding duration and higher rates of supplemental feeding among mothers using CHCs, whereas others have found no differences in these parameters [141,143,146]. Use of CHCs before 6 wk postpartum is less common, with the US Medical Eligibility Criteria for Contraceptive Use suggesting that CHCs can be used with no restrictions in breastfeeding women beyond 42 d postpartum [147]. However, several studies have observed lower exclusive breastfeeding rates and reduced breastfeeding duration even when CHCs were initiated after 42 d postpartum [148,149]. This may explain why WHO guidelines contraindicate the use of CHCs in breastfeeding mothers between 6 wk and 6 mo after birth [150]. It is advisable that in the weeks following the onset of secretory activation, progestin-only methods of contraception are used for breastfeeding women. Combined hormonal methods of contraception containing estrogen and progestin may be considered as a viable alternative ≥6 wk postpartum in breastfeeding women.

The altered pharmacokinetics observed in obesity provide a biological basis for potential changes in the metabolic effects of hormonal contraception in women with obesity [151]. For example, elevated levels of lipoprotein in women with obesity may compete with steroid contraceptives for binding sites on albumin, leading to higher concentrations of unbound forms [151]. Additionally, decreased levels of circulating sex hormone-binding globulin in women with obesity may contribute to higher levels of free and bioactive estrogens and progestins available to hormonally sensitive tissues [152,153]. Combined with higher levels of locally produced and stored steroid hormones, the use of hormonal contraceptives could potentially exert a more pronounced effect on milk production in women with obesity. Therefore, it is crucial to investigate whether the influence of hormonal contraceptives on milk production varies across populations with diverse BMI.

## Discussion

This review provides a comprehensive overview of the causes of LMS and highlights the significance of maternal obesity. It explores the roles of estrogens, progesterone, and related external factors in influencing milk production. Estrogens and progesterone contribute to mammary gland development, regulate prolactin actions, and participate in the synthesis of milk components. Maternal exposure to hormones from external sources, such as diet or environmental factors, can interact with estrogen receptors, impacting milk production with potential variations based on maternal BMI. However, the current understanding of human lactation is limited, primarily relying on animal studies and *in vitro* data. Furthermore, existing epidemiologic studies have predominantly focused on serum hormone concentrations, neglecting the important excretion route of breast milk, indicating the need for future investigations. The dose-dependent effects of exogenous hormones require further exploration, as they hold promise for potential strategies to mitigate the risks of obesity-associated LMS through low-dose interventions. Moreover, additional research is necessary to reassure the optimal timing for initiating different hormonal contraceptives in breastfeeding women. Importantly, the implications and interactions of these factors in diverse BMI populations remain to be elucidated. Advancing our understanding of lactation endocrinology through studies in these areas will provide valuable insights for the development of effective interventions aimed at optimizing milk production.

### Author contributions

The authors’ responsibilities were as follows – XJ, SLP, CTL, DTG: conceived of the study; XJ: contributed to design, data collection, and visualization; XJ, SLP, NLT, CTL, DTG: were involved in writing and editing the manuscript; and all authors: read and approved the final manuscript.

### Conflict of interest

The authors report no conflicts of interest.

### Funding

SLP, CTL, and DTG are supported by an unrestricted research grant from Medela AG, administered by The University of Western Australia. XJ is supported by The University of Western Australia (UWA) – China Scholarship Council (CSC) Joint PhD Scholarship and UWA – CSC Higher Degree by Research Top-Up Scholarship. The funders had no role in the design of the study, in the collection or interpretation of data, in the writing of the manuscript, or in the decision to publish the results.

## References

[1] Martin C.R., Ling P.R., Blackburn G.L. (2016). Review of infant feeding: key features of breast milk and infant formula. Nutrients.

[2] Geddes D.T., Gridneva Z., Perrella S.L., Mitoulas L.R., Kent J.C., Stinson L.F. (2021). 25 years of research in human lactation: from discovery to translation. Nutrients.

[3] World Health Organization Breastfeeding Recommendations. https://www.who.int/health-topics/breastfeeding#tab=tab_2.

[4] UNICEF (2022). https://data.unicef.org/topic/nutrition/breastfeeding/.

[5] Li R., Fein S.B., Chen J., Grummer-Strawn L.M. (2008). Why mothers stop breastfeeding: mothers’ self-reported reasons for stopping during the first year. Pediatrics.

[6] Radwan H. (2013). Patterns and determinants of breastfeeding and complementary feeding practices of Emirati mothers in the United Arab Emirates. BMC Public Health.

[7] Kent J.C., Ashton E., Hardwick C.M., Rea A., Murray K., Geddes D.T. (2021). Causes of perception of insufficient milk supply in Western Australian mothers. Matern. Child Nutr..

[8] Lee S., Kelleher S.L. (2016). Biological underpinnings of breastfeeding challenges: the role of genetics, diet, and environment on lactation physiology. Am. J. Physiol. Endocrinol. Metab..

[9] Marasco L.A. (2014). Unsolved mysteries of the human mammary gland: defining and redefining the critical questions from the lactation consultant’s perspective. J. Mammary Gland Biol. Neoplasia.

[10] Peaker M. (1991). Production of hormones by the mammary gland: short review. Endocr. Regul..

[11] Hannan F.M., Elajnaf T., Vandenberg L.N., Kennedy S.H., Thakker R.V. (2023). Hormonal regulation of mammary gland development and lactation. Nat. Rev. Endocrinol..

[12] Sadovnikova A., Wysolmerski J.J., Hovey R.C., Kovacs C.S., Deal C.L. (2020). Maternal-Fetal and Neonatal Endocrinology.

[13] Vandenberg L.N. (2021). Endocrine disrupting chemicals and the mammary gland. Adv. Pharmacol..

[14] Rudel R.A., Fenton S.E., Ackerman J.M., Euling S.Y., Makris S.L. (2011). Environmental exposures and mammary gland development: state of the science, public health implications, and research recommendations. Environ. Health Perspect..

[15] Veiga-Lopez A., Pu Y., Gingrich J., Padmanabhan V. (2018). Obesogenic endocrine disrupting chemicals: identifying knowledge gaps. Trends Endocrinol. Metab..

[16] Fent K., Escher C., Caminada D. (2006). Estrogenic activity of pharmaceuticals and pharmaceutical mixtures in a yeast reporter gene system. Reprod. Toxicol..

[17] Tapiero H., Nguyen Ba G., Tew K.D. (2002). Estrogens and environmental estrogens, Biomed. Pharmacother.

[18] Annamalai J., Namasivayam V. (2015). Endocrine disrupting chemicals in the atmosphere: their effects on humans and wildlife. Environ. Int..

[19] Weaver S.R., Hernandez L.L. (2016). Autocrine-paracrine regulation of the mammary gland. J. Dairy Sci..

[20] Daly S.E., Owens R.A., Hartmann P.E. (1993). The short-term synthesis and infant-regulated removal of milk in lactating women. Exp. Physiol..

[21] Jonas W., Woodside B. (2016). Physiological mechanisms, behavioral and psychological factors influencing the transfer of milk from mothers to their young. Horm. Behav..

[22] Huang S.K., Chih M.H. (2020). Increased breastfeeding frequency enhances milk production and infant weight gain: correlation with the basal maternal prolactin level. Breastfeed. Med..

[23] Pérez-Escamilla R., Hromi-Fiedler A., Rhodes E.C., Neves P.A.R., Vaz J., Vilar-Compte M. (2022). Impact of prelacteal feeds and neonatal introduction of breast milk substitutes on breastfeeding outcomes: a systematic review and meta-analysis. Matern. Child Nutr..

[24] Pérez-Escamilla R., Segura-Millán S., Canahuati J., Allen H. (1996). Prelacteal feeds are negatively associated with breast-feeding outcomes in Honduras. J. Nutr..

[25] Stevens E.E., Patrick T.E., Pickler R. (2009). A history of infant feeding. J. Perinat. Educ..

[26] Santacruz-Salas E., Aranda-Reneo I., Segura-Fragoso A., Cobo-Cuenca A.I., Laredo-Aguilera J.A., Carmona-Torres J.M. (2019). Mothers’ expectations and factors influencing exclusive breastfeeding during the first 6 months. Int. J. Environ. Res. Public Health.

[27] Garbin C.P., Deacon J.P., Rowan M.K., Hartmann P.E., Geddes D.T. (2009). Association of nipple piercing with abnormal milk production and breastfeeding. JAMA.

[28] Schiff M., Algert C.S., Ampt A., Sywak M.S., Roberts C.L. (2014). The impact of cosmetic breast implants on breastfeeding: a systematic review and meta-analysis. Int. Breastfeed. J..

[29] Kraut R.Y., Brown E., Korownyk C., Katz L.S., Vandermeer B., Babenko O. (2017). The impact of breast reduction surgery on breastfeeding: systematic review of observational studies. PLOS ONE.

[30] Ludvigsson J.F., Ludvigsson J. (2005). Socio-economic determinants, maternal smoking and coffee consumption, and exclusive breastfeeding in 10205 children. Acta Paediatr.

[31] Ladomenou F., Kafatos A., Galanakis E. (2007). Risk factors related to intention to breastfeed, early weaning and suboptimal duration of breastfeeding. Acta Paediatr.

[32] Napierala M., Mazela J., Merritt T.A., Florek E. (2016). Tobacco smoking and breastfeeding: effect on the lactation process, breast milk composition and infant development. A critical review. Environ. Res..

[33] Donath S.M., Amir L.H., ALSPAC Study Team (2004). The relationship between maternal smoking and breastfeeding duration after adjustment for maternal infant feeding intention. Acta Paediatr.

[34] Bahadori B., Riediger N.D., Farrell S.M., Uitz E., Moghadasian M.F. (2013). Hypothesis: smoking decreases breast feeding duration by suppressing prolactin secretion, Med. Hypotheses.

[35] Blake C.A., Sawyer C.H. (1972). Nicotine blocks the suckling-induced rise in circulating prolactin in lactating rats. Science.

[36] Criswell R., Crawford K.A., Bucinca H., Romano M.E. (2020). Endocrine-disrupting chemicals and breastfeeding duration: a review. Curr. Opin. Endocrinol. Diabetes Obes..

[37] Arbour M.W., Kessler J.L. (2013). Mammary hypoplasia: not every breast can produce sufficient milk. J. Midwifery Womens Health.

[38] Kam R.L., Amir L.H., Cullinane M. (2021). Is there an association between breast hypoplasia and breastfeeding outcomes? A systematic review. Breastfeed. Med..

[39] Lee S., Hennigar S.R., Alam S., Nishida K., Kelleher S.L. (2015). Essential role for zinc transporter 2 (ZnT2)-mediated zinc transport in mammary gland development and function during lactation. J. Biol. Chem..

[40] McCormick N.H., Hennigar S.R., Kiselyov K., Kelleher S.L. (2014). The biology of zinc transport in mammary epithelial cells: implications for mammary gland development, lactation, and involution. J. Mammary Gland Biol. Neoplasia..

[41] Rivera O.C., Geddes D.T., Barber-Zucker S., Zarivach R., Gagnon A., Soybel D.I. (2020). A common genetic variant in zinc transporter ZnT2 (Thr288Ser) is present in women with low milk volume and alters lysosome function and cell energetics. Am. J. Physiol. Cell Physiol..

[42] McIntyre H.D., Catalano P., Zhang C., Desoye G., Mathiesen E.R., Damm P. (2019). Gestational diabetes mellitus. Nat. Rev. Dis. Primers.

[43] Suwaydi M.A., Wlodek M.E., Lai C.T., Prosser S.A., Geddes D.T., Perrella S.L. (2022). Delayed secretory activation and low milk production in women with gestational diabetes: a case series. BMC Pregnancy Childbirth.

[44] Riddle S.W., Nommsen-Rivers L.A. (2016). A case control study of diabetes during pregnancy and low milk supply. Breastfeed. Med..

[45] Nguyen P.T.H., Pham N.M., Chu K.T., Van Duong D., Van Do D. (2019). Gestational diabetes and breastfeeding outcomes: a systematic review. Asia Pac. J. Public Health.

[46] Menzies K.K., Lee H.J., Lefèvre C., Ormandy C.J., Macmillan K.L., Nicholas K.R. (2010). Insulin, a key regulator of hormone responsive milk protein synthesis during lactogenesis in murine mammary explants. Funct. Integr. Genomics.

[47] Menzies K.K., Lefèvre C., Macmillan K.L., Nicholas K.R. (2009). Insulin regulates milk protein synthesis at multiple levels in the bovine mammary gland. Funct. Integr. Genomics.

[48] Neubauer S.H., Ferris A.M., Chase C.G., Fanelli J., Thompson C.A., Lammi-Keefe C.J. (1993). Delayed lactogenesis in women with insulin-dependent diabetes mellitus. Am. J. Clin. Nutr..

[49] Hummel S., Winkler C., Schoen S., Knopff A., Marienfeld S., Bonifacio E. (2007). Breastfeeding habits in families with Type 1 diabetes. Diabet. Med..

[50] Britten F.L., Lai C.T., Geddes D.T., Callaway L.K., Duncan E.L. (2022). Is secretory activation delayed in women with type two diabetes? A pilot study. Nutrients.

[51] Marasco L., Marmet C., Shell E. (2000). Polycystic ovary syndrome: a connection to insufficient milk supply?. J. Hum. Lact..

[52] Vanky E., Isaksen H., Haase Moen M., Carlsen S.M. (2008). Breastfeeding in polycystic ovary syndrome. Acta Obstet. Gynecol. Scand..

[53] Rassie K., Mousa A., Joham A., Teede H.J. (2021). Metabolic conditions including obesity, diabetes, and polycystic ovary syndrome: implications for breastfeeding and breastmilk composition. Semin. Reprod. Med..

[54] Carlsen S.M., Jacobsen G., Vanky E. (2010). Mid-pregnancy androgen levels are negatively associated with breastfeeding. Acta Obstet. Gynecol. Scand..

[55] Joham A.E., Nanayakkara N., Ranasinha S., Zoungas S., Boyle J., Harrison C.L. (2016). Obesity, polycystic ovary syndrome and breastfeeding: an observational study. Acta Obstet. Gynecol. Scand..

[56] Legro R.S. (2000). The genetics of obesity. Lessons for polycystic ovary syndrome. Ann. N. Y. Acad. Sci..

[57] Yogev Y., Visser G.H.A. (2009). Obesity, gestational diabetes and pregnancy outcome, Semin. Fetal Neonatal Med.

[58] Ye J. (2013). Mechanisms of insulin resistance in obesity. Front. Med..

[59] Chang Y.S., Glaria A.A., Davie P., Beake S., Bick D. (2020). Breastfeeding experiences and support for women who are overweight or obese: a mixed-methods systematic review. Matern. Child Nutr..

[60] Turcksin R., Bel S., Galjaard S., Devlieger R. (2014). Maternal obesity and breastfeeding intention, initiation, intensity and duration: a systematic review. Matern. Child Nutr..

[61] Flint D.J., Travers M.T., Barber M.C., Binart N., Kelly P.A. (2005). Diet-induced obesity impairs mammary development and lactogenesis in murine mammary gland. Am. J. Physiol. Endocrinol. Metab..

[62] Siiteri P.K. (1987). Adipose tissue as a source of hormones. Am. J. Clin. Nutr..

[63] Simpson E.R. (2000). Biology of aromatase in the mammary gland. J. Mammary Gland Biol. Neoplasia.

[64] Brown K.A. (2014). Impact of obesity on mammary gland inflammation and local estrogen production. J. Mammary Gland Biol. Neoplasia.

[65] Azziz R. (1989). Reproductive endocrinologic alterations in female asymptomatic obesity. Fertil. Steril..

[66] Knight C.H. (2020). An endocrine hypothesis to explain obesity-related lactation insufficiency in breastfeeding mothers. J. Dairy Res..

[67] Hilson J.A., Rasmussen K.M., Kjolhede C.L. (2004). High prepregnant body mass index is associated with poor lactation outcomes among white, rural women independent of psychosocial and demographic correlates. J. Hum. Lact..

[68] Garcia A.H., Voortman T., Baena C.P., Chowdhurry R., Muka T., Jaspers L. (2016). Maternal weight status, diet, and supplement use as determinants of breastfeeding and complementary feeding: a systematic review and meta-analysis. Nutr. Rev..

[69] Teixeira D., Pestana D., Santos C., Correia-Sá L., Marques C., Norberto S. (2015). Inflammatory and cardiometabolic risk on obesity: role of environmental xenoestrogens. J. Clin. Endocrinol. Metab..

[70] Darbre P.D. (2017). Endocrine disruptors and obesity. Curr. Obes. Rep..

[71] Katz K.A., Nilsson I., Rasmussen K.M. (2010). Danish health care providers’ perception of breastfeeding difficulty experienced by women who are obese, have large breasts, or both. J. Hum. Lact..

[72] Massov L. (2015). Clinically overweight and obese mothers and low rates of breastfeeding: exploring women’s perspectives. J. N. Z. Coll. Midwives..

[73] Jarlenski M., McManus J., Diener-West M., Schwarz E.B., Yeung E., Bennett W.L. (2014). Association between support from a health professional and breastfeeding knowledge and practices among obese women: evidence from the Infant Practices Study II. Womens Health Issues.

[74] Gruber C.J., Tschugguel W., Schneeberger C., Huber J.C. (2002). Production and actions of estrogens. N. Engl. J. Med..

[75] Ni Y., Chen Q., Cai J., Xiao L., Zhang J. (2021). Three lactation-related hormones: regulation of hypothalamus-pituitary axis and function on lactation. Mol. Cell. Endocrinol..

[76] Watson C.S., Jeng Y.J., Kochukov M.Y. (2008). Nongenomic actions of estradiol compared with estrone and estriol in pituitary tumor cell signaling and proliferation. FASEB J.

[77] Martin R.H., Oakey R.E. (1982). The role of antenatal oestrogen in post-partum human lactogenesis: evidence from oestrogen-deficient pregnancies. Clin. Endocrinol. (Oxf.).

[78] Feng Y., Manka D., Wagner K.U., Khan S.A. (2007). Estrogen receptor-alpha expression in the mammary epithelium is required for ductal and alveolar morphogenesis in mice. Proc. Natl. Acad. Sci. U. S. A..

[79] Tong J., Zhang H., Wu Y., Wang Y., Li Q., Liu Y. (2016). Oestrogens and prolactin regulate mammary gland epithelial cell growth by modulation of the Wnt signal pathway. Slov. Vet. Res..

[80] Chu M., Zhao Y., Yu S., Hao Y., Zhang P., Feng Y. (2018). miR-15b negatively correlates with lipid metabolism in mammary epithelial cells. Am. J. Physiol. Cell. Physiol..

[81] Chu M., Zhao Y., Yu S., Hao Y., Zhang P., Feng Y. (2018). MicroRNA-221 may be involved in lipid metabolism in mammary epithelial cells. Int. J. Biochem. Cell. Biol..

[82] Ingram J.C., Woolridge M.W., Greenwood R.J., McGrath L. (1999). Maternal predictors of early breast milk output. Acta Paediatr.

[83] Athie F., Bachman K.C., Head H.H., Hayen M.J., Wilcox C.J. (1996). Estrogen administered at final milk removal accelerates involution of bovine mammary gland. J. Dairy Sci..

[84] Agenäs S., Lundström I., Holtenius K. (2019). The effect of 17β-estradiol on lactose in plasma and urine in dairy cows in late lactation. J. Dairy Res..

[85] Yart L., Finot L., Lollivier V., Dessauge F. (2013). Oestradiol enhances apoptosis in bovine mammary epithelial cells in vitro. J. Dairy Res..

[86] Lim C.L., Or Y.Z., Ong Z., Chung H.H., Hayashi H., Shrestha S. (2020). Estrogen exacerbates mammary involution through neutrophil-dependent and -independent mechanism. eLife.

[87] Simpson E.R. (2003). Sources of estrogen and their importance. J. Steroid Biochem. Mol. Biol..

[88] Simpson E.R., Mahendroo M.S., Means G.D., Kilgore M.W., Hinshelwood M.M., Graham-Lorence S. (1994). Aromatase cytochrome P450, the enzyme responsible for estrogen biosynthesis. Endocr. Rev..

[89] Ren Z., Zhang A., Zhang J., Wang R., Xia H. (2022). Role of perinatal biological factors in delayed lactogenesis II among women with pre-pregnancy overweight and obesity. Biol. Res. Nurs..

[90] Tuckey R.C. (2005). Progesterone synthesis by the human placenta. Placenta.

[91] Beleut M., Rajaram R.D., Caikovski M., Ayyanan A., Germano D., Choi Y. (2010). Two distinct mechanisms underlie progesterone-induced proliferation in the mammary gland. Proc. Natl. Acad. Sci. U. S. A..

[92] Lain A.R., Creighton C.J., Conneely O.M. (2013). Research resource: progesterone receptor targetome underlying mammary gland branching morphogenesis. Mol. Endocrinol..

[93] Collier R.J., Tucker H.A. (1978). Regulation of cortisol uptake in mammary tissue of cows. J. Dairy Sci..

[94] Djiane J., Durand P. (1977). Prolactin–progesterone antagonism in self regulation of prolactin receptors in the mammary gland. Nature.

[95] Teyssot B., Houdebine L.M. (1981). Induction of casein synthesis by prolactin and inhibition by progesterone in the pseudopregnant rabbit treated by colchicine without any simultaneous variations of casein mRNA concentration. Eur. J. Biochem..

[96] Turkington R.W., Hill R.L. (1969). Lactose synthetase: progesterone inhibition of the induction of alpha-lactalbumin. Science.

[97] Cox D.B., Kent J.C., Casey T.M., Owens R.A., Hartmann P.E. (1999). Breast growth and the urinary excretion of lactose during human pregnancy and early lactation: endocrine relationships. Exp. Physiol..

[98] Neifert M.R., McDonough S.L., Neville M.C. (1981). Failure of lactogenesis associated with placental retention. Am. J. Obstet. Gynecol..

[99] Nguyen D.A., Parlow A.F., Neville M.C. (2001). Hormonal regulation of tight junction closure in the mouse mammary epithelium during the transition from pregnancy to lactation. J. Endocrinol..

[100] Mohammad M.A., Hadsell D.L., Haymond M.W. (2012). Gene regulation of UDP-galactose synthesis and transport: potential rate-limiting processes in initiation of milk production in humans. Am. J. Physiol. Endocrinol. Metab..

[101] Berg M.N., Dharmarajan A.M., Waddell B.J. (2002). Glucocorticoids and progesterone prevent apoptosis in the lactating rat mammary gland. Endocrinology.

[102] Herrenkohl L.R. (1972). Effects on lactation of progesterone injections administered after parturition in the rat. Proc. Soc. Exp. Biol. Med..

[103] Fehér T., Bodrogi L. (1982). A comparative study of steroid concentrations in human adipose tissue and the peripheral circulation. Clin. Chim. Acta..

[104] Hamudikuwanda H., Gallo G., Block E., Downey B.R. (1996). Adipose tissue progesterone concentrations in dairy cows during late pregnancy and early lactation. Anim. Reprod. Sci..

[105] Rasmussen K.M., Kjolhede C.L. (2004). Prepregnant overweight and obesity diminish the prolactin response to suckling in the first week postpartum. Pediatrics.

[106] Capuco A.V., Tucker H.A. (1980). Progesterone inhibition of glucocorticoid binding to mammary tissue from lactating and nonlactating cows. Proc. Soc. Exp. Biol. Med..

[107] Landete J.M., Arqués J., Medina M., Gaya P., de Las Rivas B., Muñoz R. (2016). Bioactivation of phytoestrogens: intestinal bacteria and health. Crit. Rev. Food Sci. Nutr..

[108] Viggiani M.T., Polimeno L., Di Leo A., Barone M. (2019). Phytoestrogens: dietary intake, bioavailability, and protective mechanisms against colorectal neoproliferative lesions. Nutrients.

[109] Mueller S.O., Simon S., Chae K., Metzler M., Korach K.S. (2004). Phytoestrogens and their human metabolites show distinct agonistic and antagonistic properties on estrogen receptor alpha (Eralpha) and Erbeta in human cells. Toxicol. Sci..

[110] Tsugami Y., Wakasa H., Kawahara M., Nishimura T., Kobayashi K. (2022). Isoflavones and their metabolites influence the milk production ability of bovine mammary epithelial cells in a type-specific manner. Anim. Sci. J..

[111] Tsugami Y., Suzuki N., Suzuki T., Nishimura T., Kobayashi K. (2020). Regulatory effects of soy isoflavones and their metabolites in milk production via different ways in mice. J. Agric. Food Chem..

[112] Tsugami Y., Matsunaga K., Suzuki T., Nishimura T., Kobayashi K. (2017). Isoflavones and their metabolites influence the milk component synthesis ability of mammary epithelial cells through prolactin/STAT5 signaling. Mol. Nutr. Food Res..

[113] Tsugami Y., Matsunaga K., Suzuki T., Nishimura T., Kobayashi K. (2017). Phytoestrogens weaken the blood–milk barrier in lactating mammary epithelial cells by affecting tight junctions and cell viability. J. Agric. Food Chem..

[114] Kumai A., Tsugami Y., Wakasa H., Suzuki N., Suzuki T., Nishimura T. (2020). Adverse effects of coumestrol and genistein on mammary morphogenesis and future milk production ability of mammary epithelial cells. Adv. Biosyst..

[115] Cederroth C.R., Vinciguerra M., Kühne F., Madani R., Doerge D.R., Visser T.J. (2007). A phytoestrogen-rich diet increases energy expenditure and decreases adiposity in mice. Environ. Health Perspect..

[116] Kuryłowicz A., Cąkała-Jakimowicz M., Puzianowska-Kuźnicka M. (2020). Targeting abdominal obesity and its complications with dietary phytoestrogens. Nutrients.

[117] Dang Z.C., Lowik C. (2005). Dose-dependent effects of phytoestrogens on bone. Trends Endocrinol. Metab..

[118] Talaei M., Pan A. (2015). Role of phytoestrogens in prevention and management of type 2 diabetes. World J. Diabetes.

[119] Iino C., Shimoyama T., Iino K., Yokoyama Y., Chinda D., Sakuraba H. (2019). Daidzein intake is associated with equol producing status through an increase in the intestinal bacteria responsible for equol production. Nutrients.

[120] Usui T., Tochiya M., Sasaki Y., Muranaka K., Yamakage H., Himeno A. (2013). Effects of natural S-equol supplements on overweight or obesity and metabolic syndrome in the Japanese, based on sex and equol status. Clin. Endocrinol. (Oxf.).

[121] Alshannaq A., Yu J.H. (2017). Occurrence, toxicity, and analysis of major mycotoxins in food. Int. J. Environ. Res. Public Health.

[122] Kinkade C.W., Rivera-Núñez Z., Gorcyzca L., Aleksunes L.M., Barrett E.S. (2021). Impact of fusarium-derived mycoestrogens on female reproduction: a systematic review. Toxins (Basel).

[123] Mauro T., Hao L., Pop L.C., Buckley B., Schneider S.H., Bandera E.V. (2018). Circulating zearalenone and its metabolites differ in women due to body mass index and food intake. Food Chem. Toxicol..

[124] Memiş E.Y., Yalçın S.S. (2021). Human milk mycotoxin contamination: smoking exposure and breastfeeding problems. J. Matern. Fetal Neonatal Med..

[125] Wu K., Jia S., Xue D., Rajput S.A., Liu M., Qi D. (2022). Dual effects of zearalenone on aflatoxin B1–induced liver and mammary gland toxicity in pregnant and lactating rats. Ecotoxicol. Environ. Saf..

[126] Gao X., Sun L., Zhang N., Li C., Zhang J., Xiao Z. (2017). Gestational zearalenone exposure causes reproductive and developmental toxicity in pregnant rats and female offspring. Toxins (Basel).

[127] Yip K.Y., Wan M.L.Y., Wong A.S.T., Korach K.S., El-Nezami H. (2017). Combined low-dose zearalenone and aflatoxin B1 on cell growth and cell-cycle progression in breast cancer MCF-7 cells. Toxicol. Lett..

[128] González-Alvarez M.E., McGuire B.C., Keating A.F. (2021). Obesity alters the ovarian proteomic response to zearalenone exposure. Biol. Reprod..

[129] Frizzell C., Uhlig S., Miles C.O., Verhaegen S., Elliott C.T., Eriksen G.S. (2015). Biotransformation of zearalenone and zearalenols to their major glucuronide metabolites reduces estrogenic activity. Toxicol. In Vitro.

[130] Hong H., Tong W., Fang H., Shi L., Xie Q., Wu J. (2002). Prediction of estrogen receptor binding for 58,000 chemicals using an integrated system of a tree-based model with structural alerts. Environ. Health Perspect..

[131] Lallas P.L. (2001). The Stockholm Convention on persistent organic pollutants. Am. J. Int. Law.

[132] Mehlsen A., Høllund L., Boye H., Frederiksen H., Andersson A.M., Bruun S. (2022). Pregnancy exposure to bisphenol A and duration of breastfeeding. Environ. Res..

[133] Kasper N., Peterson K.E., Zhang Z., Ferguson K.K., Sánchez B.N., Cantoral A. (2016). Association of bisphenol A exposure with breastfeeding and perceived insufficient milk supply in Mexican women. Matern. Child Health J..

[134] Altamirano G.A., Muñoz-de-Toro M., Luque E.H., Gómez A.L., Delconte M.B., Kass L. (2015). Milk lipid composition is modified by perinatal exposure to bisphenol A. Mol. Cell. Endocrinol..

[135] Kass L., Altamirano G.A., Bosquiazzo V.L., Luque E.H., Muñoz-de-Toro M. (2012). Perinatal exposure to xenoestrogens impairs mammary gland differentiation and modifies milk composition in Wistar rats. Reprod. Toxicol..

[136] LaPlante C.D., Catanese M.C., Bansal R., Vandenberg L.N. (2017). Bisphenol S alters the lactating mammary gland and nursing behaviors in mice exposed during pregnancy and lactation. Endocrinology.

[137] Lee K.Y., Shibutani M., Takagi H., Kato N., Takigami S., Uneyama C. (2004). Diverse developmental toxicity of di-n-butyl phthalate in both sexes of rat offspring after maternal exposure during the period from late gestation through lactation. Toxicology.

[138] Rosen-Carole C.B., Auinger P., Howard C.R., Brownell E.A., Lanphear B.P. (2017). Low-level prenatal toxin exposures and breastfeeding duration: a prospective cohort study. Matern. Child Health J..

[139] Diamanti-Kandarakis E., Bourguignon J.P., Giudice L.C., Hauser R., Prins G.S., Soto A.M. (2009). Endocrine-disrupting chemicals: an Endocrine Society scientific statement. Endocr. Rev..

[140] Remillard R.B.J., Bunce N.J. (2002). Linking dioxins to diabetes: epidemiology and biologic plausibility. Environ. Health Perspect..

[141] Tepper N.K., Phillips S.J., Kapp N., Gaffield M.E., Curtis K.M. (2016). Combined hormonal contraceptive use among breastfeeding women: an updated systematic review. Contraception.

[142] Phillips S.J., Tepper N.K., Kapp N., Nanda K., Temmerman M., Curtis K.M. (2016). Progestogen-only contraceptive use among breastfeeding women: a systematic review. Contraception.

[143] Lopez L.M., Grey T.W., Stuebe A.M., Chen M., Truitt S.T., Gallo M.F. (2015). Combined hormonal versus nonhormonal versus progestin-only contraception in lactation. Cochrane Database Syst. Rev..

[144] Stanton T.A., Blumenthal P.D. (2019). Postpartum hormonal contraception in breastfeeding women. Curr. Opin. Obstet. Gynecol..

[145] Sothornwit J., Kaewrudee S., Lumbiganon P., Pattanittum P., Averbach S.H. (2022). Immediate versus delayed postpartum insertion of contraceptive implant and IUD for contraception. Cochrane Database Syst. Rev..

[146] Kapp N., Curtis K.M. (2010). Combined oral contraceptive use among breastfeeding women: a systematic review. Contraception.

[147] Floyd S. (2020). Postpartum contraception options. Obstet. Gynecol. Clin. North Am..

[148] Nilsson S., Mellbin T., Hofvander Y., Sundelin C., Valentin J., Nygren K.G. (1986). Long-term follow-up of children breast-fed by mothers using oral contraceptives. Contraception.

[149] Peralta O., Díaz S., Juez G., Herreros C., Casado M.E., Salvatierra A.M. (1983). Fertility regulation in nursing women: V. Long-term influence of a low-dose combined oral contraceptive initiated at day 90 postpartum upon lactation and infant growth. Contraception.

[150] Grandi G., Del Savio M.C., Tassi A., Facchinetti F. (2023). Postpartum contraception: a matter of guidelines. Int. J. Gynaecol. Obstet. Published online June.

[151] Simmons K.B., Edelman A.B. (2016). Hormonal contraception and obesity. Fertil. Steril..

[152] Stanczyk F.Z., Grimes D.A. (2008). Sex hormone-binding globulin: not a surrogate marker for venous thromboembolism in women using oral contraceptives. Contraception.

[153] Hautanen A. (2000). Synthesis and regulation of sex hormone-binding globulin in obesity. Int. J. Obes. Relat. Metab. Disord..

